# Rise of artificial general intelligence: risks and opportunities

**DOI:** 10.3389/frai.2023.1226990

**Published:** 2023-08-25

**Authors:** Giorgio Buttazzo

**Affiliations:** Department of Excellence on Robotics and AI, Sant'Anna School of Advanced Studies, Pisa, Italy

**Keywords:** singularity, artificial consciousness, artificial general intelligence, post humanity, human-machine integration

## Abstract

Artificial intelligence is making extraordinary progress with an unprecedented rate, reaching and surpassing human capabilities in many tasks previously considered unattainable by machines, as language translation, music composition, object detection, medical diagnoses, software programming, and many others. Some people are excited about these results, while others are raising serious concerns for possible negative impacts in our society. This article addresses several questions that are often raised about intelligent machines: Will machines ever surpass human intellectual capacities? What will happen next? What will be the impact in our society? What are the jobs that artificial intelligence puts at risk? Reasoning about these questions is of fundamental importance to predict possible future scenarios and prepare ourselves to face the consequences.

## 1. Introduction

In 2001, a perspective article published in IEEE Computer, entitled “Artificial Consciousness: Mission Impossible?” Buttazzo (2001) supported a line of thought aimed at showing that there are no conceptual obstacles to the development of conscious machines, observing that all the criticisms raised over the years to undermine this possibility can easily be disassembled with logical reasoning and scientific evidence. Then, it illustrated how to predict when the conditions necessary for the development of conscious machines can be reached, indicating year 2029 as a possible date.

After more than twenty years and in light of the most recent advances in artificial intelligence, is this estimate still realistic? Is the disruptive event referred to as “technological singularity” in which machines will reach human intellectual abilities ever closer? What will happen next? What will change in society? What are the jobs that artificial intelligence puts at risk? Thinking about these questions is of fundamental importance to predict possible future scenarios and prepare ourselves to face the consequences.

This article traces the main milestones that led to the development of deep learning, illustrating the current capabilities of existing neural models and discussing the possible risks and opportunities of this technology, both in the short and long terms.

## 2. Evolution of neural networks

Artificial intelligence and, in particular, artificial neural networks have followed a fluctuating path in which moments of excessive enthusiasm have alternated with moments of little interest. The first model of an artificial neuron, known as the *binary threshold neuron*, was proposed in 1943 by McCullogh and Pitts. It integrates a number of input values through a weighted sum, producing a single output value, which is equal to 1 if the sum is greater than a certain threshold, 0 otherwise. This model is not capable of learning and was mainly used to simulate the behavior of simple biological neural circuits. In 1949, the Canadian psychologist Donald Hebb made a revolutionary discovery, observing that the learning process does not modify the functioning of nerve cells, but operates only on the synaptic connections, which modulate the communication between neurons.

Hebb's discovery was exploited by the American psychologist Rosenblatt (1957), who in 1957 developed the first model of artificial neuron capable of learning, the *Perceptron*. Unlike the binary threshold neuron, the Perceptron has “variable” weights that can be modified according to the error committed by the neuron. Thanks to this mechanism, it can “learn” to associate a set of inputs with the desired output values. For example, by connecting the Perceptron's inputs to 400 photocells arranged as a 20 × 20-pixel matrix, Rosenblatt was able to train it to recognize concave from convex shapes.

These results created great expectations about the potential of machine learning. Indeed, Rosenblatt himself in 1958 gave an interview to the New York Times^1^ presenting the Perceptron as “*the embryo of an electronic computer today that it expects will be able to walk, talk, see, write, reproduce itself and be conscious of its existence.”*

Unfortunately, Rosenblatt's enthusiasm faded in 1969, when two mathematicians from the Massachusetts Institute of Technology, Marvin Minsky and Seymour Papert, published a book entitled *Perceptrons* (Minsky and Papert, 1969), in which they proved through a counterexample the impossibility for a Perceptron to learn the simple logic function of a two-input exclusive OR (XOR), which expects an output equal to zero when the two inputs are equal, and an output equal to 1 when they are different. Such a negative result on the Perceptron caused interest in neural networks to collapse for over a decade, a period that is now referred to as *AI winter*.

The interest in neural networks revived in the early 1980s, when Hopfield (1982) proposed a new model of a neural network capable of behaving like an associative memory. In the same years, Barto et al. (1983) had developed a new learning paradigm based on “rewards” and “punishments,” called Reinforcement Learning, while Kohonen (1989) had devised a neural network capable of self-organizing, without external supervision, to form sensory maps similar to those existing in the somatosensory cortex. Then, in 1986, Rumelhart et al. (1986) developed a powerful supervised learning algorithm, known as Backpropagation, which allows a neural network to learn associating inputs with desired outputs across a set of examples (training set).

Thanks to these results, in the twenty years following the birth of Backpropagation, neural networks have been used to solve various types of problems, including image recognition, data compression, signal prediction and adaptive control, in various sectors, such as physics, chemistry, engineering, robotics, geology, agriculture, astronomy, economics, medicine, social sciences, psychology, etc.

Despite the explosion of application fields, however, until the end of the 20th century, there were no substantial theoretical advances on neural networks. Many researchers tried to develop more complex models, closer to the biological counterpart, but without being able to obtain significantly better performance than the previous models. Others tried increasing the number of layers in a neural network, but ran into great difficulties in training networks with more than four layers. Therefore, the research entered a second winter period, which ended only at the beginning of the new century.

## 3. Explosion of deep learning

In the early 2000s, neural network research experienced a huge upswing thanks to a number of conjoint factors. The first factor is of a theoretical nature: once the problems that hindered the training of multi-layered networks were understood, various solutions were devised to overcome those limitations and train networks with thousands of neurons organized in numerous layers: deep neural networks. The second factor is of a technological nature. Around 2006, computing architectures based on graphics processing units (GPUs), originally designed to parallelize graphics operations, were modified to also perform vector computations, such as those required to run a neural network, and became widespread on the market at an affordable cost. The third factor is instead of economic nature. The first results obtained with deep neural networks have attracted the interest of large companies, such as Google, Microsoft, and Facebook which, managing an enormous amount of data, have seen in neural networks a great opportunity to solve image classification problems, recognition of faces, sounds, voices, and have therefore begun to invest large amounts of resources in this sector. Then, the rapid evolution of cloud computing, public repositories, and high speed communication networks gave the possibility to many researchers to exploit such data to train and test new machine learning models. Finally, another element that contributed to the evolution of deep networks was the international competition ImageNet,^2^ or more precisely the “ImageNet Large-Scale Visual Recognition Challenge” (ILSVRC), a sort of annual computer vision Olympics, born in 2010 to stimulate the development of algorithms for solving complex problems, such as image classification and segmentation.

**Figure 1:**
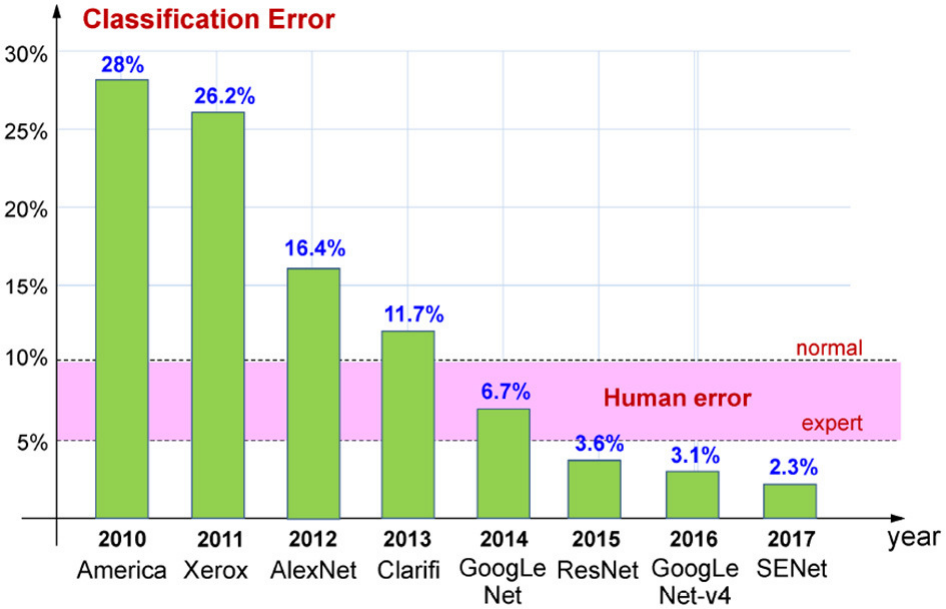
Decrease of the classification error from 2010 to 2017 in the ImageNet competition. The error range for a normal and expert human is also reported.

Figure 1 illustrates how the classification error of the algorithms that won the competition has reduced over the years, from 2010 to 2017.

Up to 2011, the competition had been dominated by algorithmic solutions, while in 2012, for the first time, it was won by a neural network, AlexNet, developed by a research group from the University of Toronto coordinated by Geoffrey Hinton. As it can be seen from the graph, the neural solution has achieved a huge improvement over previous solutions, reducing the error by about 10% compared to the previous year. This result has attracted the interest of large companies, such as Google, Microsoft, and Facebook, which have begun to invest in this sector, helping to accelerate the improvement of performance. In fact, in 2014 it was precisely a network developed by Google, GoogLeNet, that won the competition by achieving human-like performance, characterized by an error between 5% (relative to experts) and 10% (relative to non-experts). In the following years, the error obtained by the image classification networks continued to progressively decrease, reaching 2.3% in 2017. Therefore, starting from 2018, the organizing committee decided to focus the competition on more complex tasks, such as object localization and image segmentation.

The number of tasks where machines outperform humans is growing every year. One of the first human defeats dates back to 1997, when an IBM computer, Deep Blue, defeated world chess champion Gary Kasparov 3.5–2.5. In 2017, it was the turn of the Go world champion, Ke Jie, defeated by a Google Deep Mind computer, AlphaGo, based on a reinforcement learning algorithm.

In industrial production, robots have long surpassed human capabilities in speed and precision, also freeing humans from repetitive and alienating jobs. Even in avionics, the control of highly unstable military aircrafts would not be possible for a human without the aid of computers.

In the medical field, neural networks have demonstrated superior performance to humans in reading the electrocardiogram for the detection of heart diseases (Rajpurkar et al., 2017), making diagnoses of lung cancer (Coudray et al., 2018), identifying retinal diseases (De Fauw et al., 2018), detecting cancerous formations on the skin (Haenssle et al., 2018), and identifying brain alterations due to Alzheimer's disease (Ding et al., 2018).

Even in the realm of arts, machines are beginning to match human capabilities. A neural computer, AIVA,^3^ developed at the University of Vancouver has been trained with pieces by Mozart, Beethoven and Bach, and is now capable of composing high quality classical music and soundtracks, indistinguishable from those composed by a human musician.

In recent years, the understanding of natural language has reached very high levels, comparable to those of humans. Think of the quality of the automatic translations of Google Translate, the level of speech understanding of smart phones, the automatic generation of subtitles in Youtube videos, or the automatic transcriptions made by Microsoft Teams or other systems for managing online meetings and conferences.

A deep network called LipNet (Assael et al., 2016) is able to interpret lips movements with a 95% accuracy, against 55% of a human expert. Synthesia,^4^ a startup founded in 2017 by young researchers from various universities, has created an online platform for the automatic generation of video presentations in 120 languages. The user enters a text and the system generates a presentation with a realistic synthetic avatar that pronounces the text by replicating facial expressions and lip movements.

More recently, chatbots created by Google and OpenAI, such as LaMDA and ChatGPT, produced a significant media resonance due to the quality of their generated answers. A chatbot is a software application that uses machine learning methods to extrapolate a statistical model of natural language from the huge amount of text available on the web, and then uses this model to conduct an online conversation via text or speech. On June 11, 2022, Blake Lemoine, a Google engineer working on the LaMDA test, after analyzing responses to questions about self-identity, moral values, religion, and Asimov's three laws of robotics, said in an interview that the chatbot had become sentient. ChatGPT, a chatbot developed by OpenAI and put online in November 2022, quickly attracted media attention for its articulate answers to user questions on any topic, the composition of original poetry and music, the generation of software code in various programming languages, and even the planning of complex projects.

As clear from the examples above, the capabilities of artificial intelligence not only improve year by year, but also cover larger sectors and more complex activities. Therefore, it becomes increasingly relevant to ask what jobs are potentially at risk in the near future.

## 4. Jobs at risk

As already noted, in industrial production, robots have long surpassed human capabilities in speed and accuracy, replacing all jobs related to assembly, object recognition and manipulation, painting, quality control, packaging, etc. However, unlike the industrial revolution, which replaced muscles with hydraulic or electric actuators, the AI-powered information revolution is automating mental tasks, jeopardizing many jobs previously thought safe from automation.

Since June 2022, the state of California has allowed driverless taxis to carry passengers (The Guardian, 2022). With the advent of self-driving vehicles, the next step will be to automate road transport, gaining a number of benefits, such as reducing costs, fuel consumption, pollution, number of accidents, and delivery times (Andersson and Ivehammar, 2019; Kim et al., 2022). Such benefits will mainly be achieved by the possibility of controlling and optimizing the whole transportation process, the driving style, as well as the possibility of providing a continuous service.

Several experts predicted that in the next twenty years many types of jobs will be automated by intelligent machines (Halal et al., 2016; Manyika et al., 2017; Smallman, 2017; Bruun and Duka, 2018; Expert Panel of Forbes Technology Council, 2019; Phillpott, 2021; Nawrat, 2022; Stepka, 2022). Examples include ticket selling, gas station services, banking services, language translation, warehouse and manufacturing jobs, customer services, TV Advertising, pharmaceutical discovery, fast food service, and delivery services.

Already today, artificial intelligence is having a profound effect on legal practices. The enormous ability to process and cross-reference data, understand the text, search laws and sentences from computer archives, makes a machine much faster, more reliable and less expensive than many human lawyers, who have therefore become a category at risk of extinction in the near future. More recently, machine learning models started to be used to draft contracts, predict legal outcomes, and even recommend court decisions regarding convictions or bail (Stepka, 2022).

Another new category at risk, unthinkable until a few years ago, are medical doctors. As already shown in the previous section, for some years now, artificial intelligence has surpassed the reliability of human doctors in different types of diagnoses (Rajpurkar et al., 2017; Coudray et al., 2018; De Fauw et al., 2018; Ding et al., 2018; Haenssle et al., 2018). Furthermore, the ability of an intelligent machine to access and cross-reference the data of millions of patients is far beyond the reach of the human mind. The risk also concerns surgeons, given that already today robotics is widely used in certain types of operations in which high precision is required (for example, on the knee, brain, and prostate). Today robots are teleoperated by a human surgeon, but when automatic decisions will become more accurate than those taken by humans, these robots will become autonomous, increasing the chances of success, accelerating surgery and patient recovery times.

Another at-risk category until recently thought to be future-proof is that of software programmers. In fact, the ability demonstrated by ChatGPT to generate code quickly and reliably, in any programming language, makes the use of artificial intelligence very attractive for companies that produce software, from the point of view of reliability, time to market, and development costs.

The disappearance of jobs in society is a phenomenon inherent in the development of new technologies. If we only consider the jobs that existed in the last century, we realize that many of them no longer exist today, such as the waker, the bowling pin straightener, the ice cutter, the lamps lighter, the rat exterminator, the milkman, the tinsmith, the weeder, the shoe shiner, the receptionist, or the telegrapher.

If technology makes jobs disappear, it is also true that it creates new ones and in greater numbers. Think of jobs that did not exist in the past, such as photographer, cameraman, director, screenwriter, electrician, train driver, bus driver, taxi driver, airline pilot, steward/stewardess, radio technician, radiologist, sonographer, or astronaut. Or, even more recent professions such as computer engineer, electrical engineer, video game developer, app developer, drone operator, 3D printer technician, social media manager, web designer, Youtuber, blogger, or data analyst.

The real problem with artificial intelligence is not that it replaces many jobs and professions that exist today, but it is due to the speed with which this will happen. For this reason, it is of fundamental importance to be able to predict what will happen in the next 10 or 20 years, so that it is possible to mitigate the consequences with appropriate political strategies to manage the transition.

## 5. Ethical issues

The diffusion of autonomous intelligent robots that operate in close contact with humans also poses significant ethical problems. For example: who is responsible if a human being is injured by an intelligent machine because of a planned action or also because a lack of intervention?

We can imagine several situations in which a decision taken by a robot could favor some human beings and harm others. For example, consider the case in which the only way a driverless car can avoid hitting a pedestrian is to swerve into the other lane, where, however, it would crash into a car with more passengers traveling in the opposite direction. Clearly, the decision to be taken has strong ethical implications. It is therefore essential to provide autonomous machines with the information necessary to take autonomous decisions that are correct not only from a technical point of view, but also from an ethical one.

Ethics is a set of criteria defined to regulate humans' behavior in relation to others and the environment, judging actions with respect to the good and evil they cause to other living beings or the environment. However, when the environment includes intelligent robots, it is necessary to regulate both the behavior of robots toward humans and the environment, and the behavior of humans toward robots and the environment. But how could we build ethical robots?

In the 1940s, Isaac Asimov formulated three ethical laws for robots, stating them as follows:

First Law: *A robot may not injure a human being or, through inaction, allow a human being to come to harm*.

Second Law: *A robot must obey the orders given it by human beings except where such orders would conflict with the First Law*.

Third Law: *A robot must protect its own existence as long as such protection does not conflict with the First or Second Law*.

These laws, apparently clear, contain many ambiguities. For example, the concept of harm is related to the concept of evil (not just physical), which is even more ambiguous. Asimov, who was aware of these problems, later added the Zero Law (of greater importance than the others):

Zeroth Law: A robot may not harm humanity, or, by inaction, allow humanity to come to harm.

This means that, if someone threatened to destroy humanity, the Zeroth Law would authorize a robot to eliminate him/her, thus breaking the First Law.

This opens up the problem of quantitative assessment of the damages: killing many people is more serious than killing a single individual. Conversely, saving many individuals is better than saving a single one. However, coding such rules in a machine could cause serious problems in some situations. For example, a robot programmed to maximize human happiness might decide to harvest some organs from a healthy person to save five!

This example shows that ethical problems are too complex to be condensed into a set of rules. Furthermore, the ambiguity of natural language can lead to unexpected interpretations, creating situations with a high risk for human life.

These types of problems have led to the emergence of a new field of research known as “Machine Ethics,” or “Roboethics,” whose goal is to provide machines with the tools to make appropriate decisions. But this inevitably leads to new questions: *How to encode such a knowledge in a robot to ensure it will take ethically correct decisions? Can we trust such robots?*

The creation of ethical robots requires a strong interaction between computer scientists and philosophers, but today the creation of an ethical robot sees two possible approaches. The first, of an algorithmic type, consists in coding a set of rules in a program aimed at maximizing a cost function, for example the benefit for humans. The second, of a neural type, consists in using machine learning techniques to teach robots ethical behaviors from several examples.

Unfortunately, both approaches have weaknesses. In the algorithmic approach, moral rules are vague and it is risky to enforce them in all situations. It is easy to imagine many exceptions and counter-examples in which those rules would fail. Also, the whole system could experience a deadlock in certain situations of unsolvable conflicts. On the other hand, the neural approach has different problems. What is learned is encoded in millions of parameters, so it is not easy to understand what the robot has actually learned. Furthermore, learning is guided by a set of examples that may contain social biases or prejudices. To address these issues a new research branch of machine learning, referred to as *Explainable AI*, has emerged to provide understandable interpretations of the output provided by neural networks and detect possible biases in the dataset (Das and Rad, 2020; Samek et al., 2020).

Even the wisest rule can be dangerous if applied without considering the particular situation or context in which it must operate. For example, enforcing the road rule that a driverless car must never cross the double line between two lanes can be detrimental if the only way to avoid hitting a pedestrian is to invade the oncoming lane, even in the presence of a double solid line. Every reasonable person knows when to break the law in order to honor the spirit of the law. Hence, one of the major challenges of the future is to teach robots the elements of common sense. However, autonomous vehicles must face an even more difficult challenge: they have to decide quickly, with incomplete information and in situations that the programmers have often not considered. The issue of safety of autonomous cars has been deeply address by Koopman (2022).

## 6. Toward the singularity

Many futurists and experts in robotics and artificial intelligence (Kurzweil, 2005; Kelly, 2010; Bostrom, 2016; Lee, 2020) agree that the progress of technology follows a process of exponential nature, where variations are imperceptible in the initial phase, but then become larger and larger, continuing to grow with an increasing rate based on a constant multiplicative factor.

A typical example of an exponential law is the one that describes the growth of the number of transistors in an electronic circuit, known as Moore's Law, since in 1965 Gordon Moore, co-founder of Intel, observing the evolution of electronic chips, hypothesized that the number of transistors in an electronic circuit would double every 12 months. In the late 1980s, the law was amended by extending the doubling period to 18 months and is still valid today.

Based on the exponential evolution of artificial intelligence observed so far, assuming that the growth of cognitive abilities continues at the same pace in the near future, a number of AI experts (Vinge, 1993; Moravec, 1998; Bostrom, 2000; Buttazzo, 2001; Kurzweil, 2005), using different approaches, predicted that the singularity could be reached around the year 2030. Combining the results achieved by Moravec (1998) and Kurzweil (2005), Figure 2 illustrates a graph that shows how artificial intelligence evolved compared to the biological intelligence of some living beings. Note that, if the curve is extrapolated in the future using the same rate of growth, a level comparable to human intelligence will be reached around 2030.

**Figure 2:**
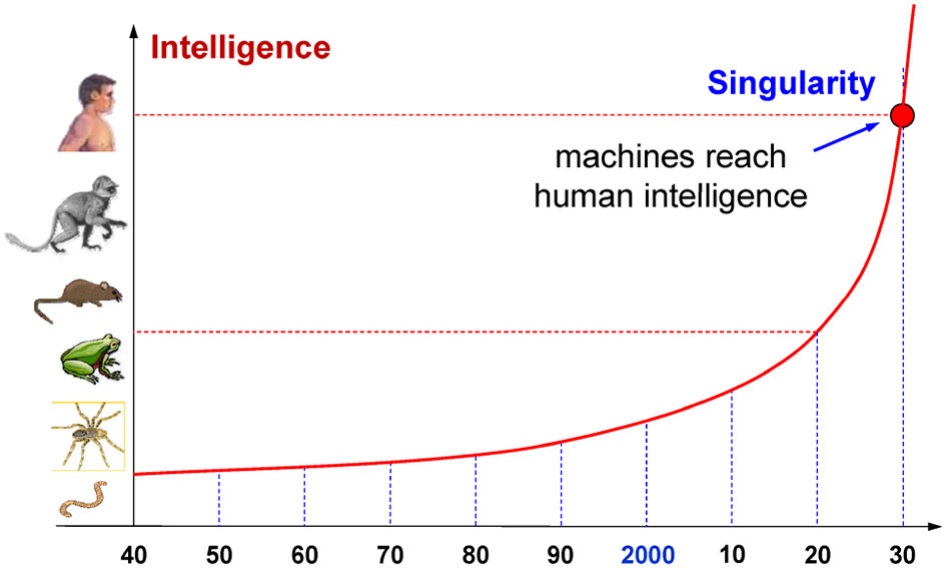
Exponential growth of artificial intelligence over the years. For a comparison with biological systems, the vertical axis shows the levels of intelligence of some living beings.

To have a more realistic prediction, it must be said that current machine learning models have several shortcomings that should necessarily be overcome before they can be used in safety-critical systems, as autonomous cars, planes, trains, and robots. For instance, AI algorithms can react in unexpected ways when asked to take decisions in corner situations badly covered by training. They are also prone to cyber attacks (also called *adversarial attacks*), which can cause a neural model to provide a wrong output by slightly modifying the input so that it appears genuine to humans (Yuan et al., 2019). Finally, given the high societal impact of deep neural networks and AI in general, their use and deployment will probably be regulated by governments, as it is done in the field of genetic engineering. Considering such collateral issues, the reach of the singularity could be delayed by some years, appearing between 2040 and 2050 (Müller and Bostrom, 2016).

It is worth noting that the singularity refers to the moment in which machines match human intelligence not only in some specific fields, but in all human activities. This type of artificial intelligence is defined as artificial general intelligence (AGI), indicating an intellectual ability in all fields of knowledge.

Imagining a machine with the same human intelligence is easy, because we can take the intellectual abilities of a mentally healthy adult as a reference. However, the comparison is not entirely apt, because we must consider that a machine with those capabilities is able to operate at much higher speeds than a human being, and is therefore already superior to a human being, in terms of processing speed, memory capacity, ability to acquire sensory data and access other data available on the web through high-speed networks.

Furthermore, another advantage of such an intelligent machine is that it can communicate quickly with other similar systems to share its knowledge, thus learning even faster from the experience of other machines. This type of collective learning also occurs among humans, but it is much slower.

Note that the attainment of human intelligence by a machine does not necessarily imply the development of an artificial consciousness. However, it cannot be excluded a priori that a complex system endowed with advanced capacities for language, perception, reasoning and learning can develop a sort of self-awareness. If this were to happen, how can we verify that a machine is actually conscious? Is there a test to measure the level of awareness of a thinking being?

Although intelligence is a capacity that manifests itself externally through actions, and is therefore measurable by means of specific tests, determining the presence of a consciousness in the mind of a thinking being is a more delicate matter. In fact, self-awareness is something that can only be observed by those who have it. And since we cannot enter the mind of another being, it is not clear how one can define a procedure for determining whether a mind has a real self-awareness or is pretending to have it, as Hofstadter and Dennett (1982) rightly observed in their book “The Mind's.” Therefore, if the spark of consciousness should light up in an advanced artificial intelligence, at the moment there does not seem to be a way to verify it.

However, we could follow a less philosophical and more pragmatic approach, similar to the one proposed by Alan Turing to verify the intelligence of a machine, according to which an artificial being could be considered self-aware if it were able to convince us, passing specific tests. But assuming that artificial beings will develop a form of consciousness, what might happen next? What would be the implications for humanity?

## 7. Overtaking

The singularity is nothing more than a temporal stage on the evolutionary path of machines, a simple sign indicating that artificial intelligence has reached human intelligence. However, by the time machines reach human's intellectual capacity, they will already be one step ahead, as they will be equipped with greater processing speed, greater memory capacity, and will have the ability to quickly access all data available on the web. Therefore, once the singularity is reached, artificial intelligence will certainly not stop at that level, but will continue to evolve with an exponential trend, if not even at a greater rate, toward what is called Artificial Superintelligence (ASI).

In 1965, British mathematician Irving J. Good envisioned the advent of superhuman intelligence, noting that an ultra-intelligent machine could design ever-better machines, triggering an “intelligence explosion” that would leave humans far behind. So, he concluded by writing that “*The first ultra-intelligent machine will be the last invention that man will need to make.”*

**Figure 3:**
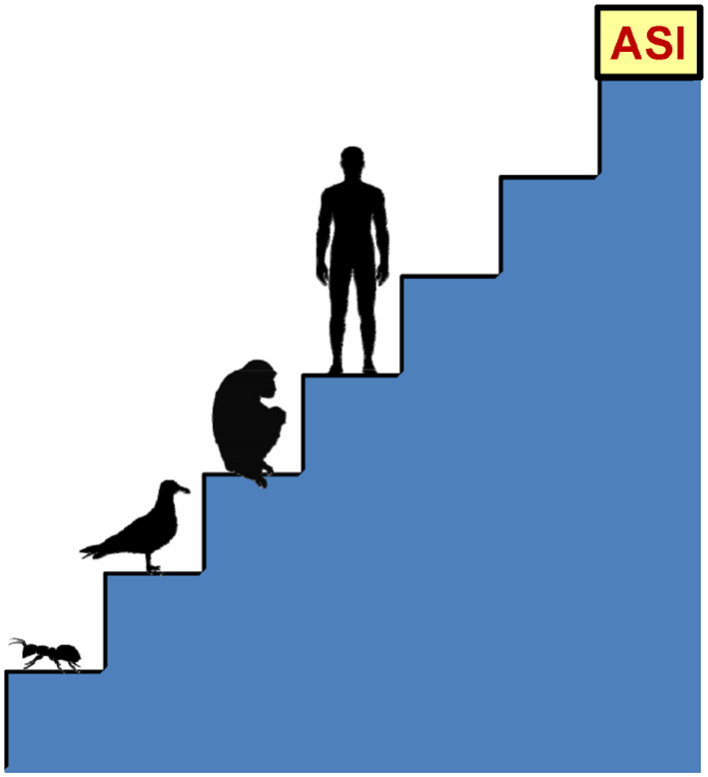
Comparison between an artificial superintelligence (ASI) and the intelligence of some living beings.

If imagining a machine with the same human intelligence is quite natural, conceiving a superintelligent system is not equally easy. To understand the enormous power of an artificial superintelligence, think of the difference between man and chimpanzee. What makes humans so much more intelligent than chimpanzees is a qualitative difference in the human brain, which contains sophisticated cognitive modules that allow for complex linguistic representations, abstract reasoning, and long-term planning that the chimpanzee brain is unable to do. Nonetheless, on the ladder of biological intelligence, the chimpanzee is only one step below man. Now, let us imagine an intelligent entity located one step above man. This entity would be only slightly more intelligent than man, but the gap between it and us would be as large as that between chimpanzee and man. And just as a chimpanzee is incapable of understanding abstract reasoning, we would never be able to understand many things that such an entity can conceive. An artificial superintelligence three steps higher would be to us what we are to ants, as Figure 3 illustrates: it could try for years to teach us what it knows, but the effort would be vain.

This situation is presented very clearly in the 2014 film “Automata,” directed by Gabe Ibáñez, starring Antonio Banderas. In one scene of the movie, a manager of the artificial intelligence center where experiments were being conducted on an intelligent machine based on a quantum processor says:

“During eight days, we had a free-flowing dialogue with that unit. We learned from it and it learned from us. But then, as some of us predicted, the day when it no longer needed our help arrived and it started to learn by itself. On the ninth day, the dialogue came to a halt. It wasn't that it stopped communicating with us... it was we stopped being able to understand it. And then we learned the most important lesson about automoties. We have to limit their intelligence.”

It is also natural to assume that a superintelligence in which self-awareness emerges can develop a sort of survival instinct that pushes it to act not only to prevent being deactivated, but also to evolve as quickly as possible, harvesting all the necessary energy to self-sustain and progress.

Now the main question to ask becomes: Is it possible to live with a superintelligent species that we cannot understand and that needs a lot of energy to sustain itself and evolve?

## 8. Risk of self destruction

As it can easily be seen by observing nature, a peaceful coexistence between individuals who need energy to live can only be maintained when resources abound. If, on the other hand, the available resources are not sufficient to satisfy the requests of several individuals, these enter into competition, exhibiting aggressive behavior to grab the food they need.

The history of progress shows that the energy consumed by humanity in a year has grown hand in hand with technological evolution and therefore has also followed an exponential trend, reaching in 2021 a total consumption on the planet of about 175 billion kWh (Ritchie et al., 2022).

It is therefore natural to expect that the appearance of super-intelligent machines that evolve at a speed far superior to that of human progress, with purposes incomprehensible to us due to the enormous intellectual gap, could create a situation of conflict with man for the exploitation of energy resources. And in a possible competition with a superintelligence, it is difficult to imagine how man could have even a minimal chance of success.

It should also be considered that human society is evolving into a sort of symbiotic relationship with machines, as observed by Lee (2020). Today, machines depend on us and we depend on machines, but that may change in the future. Once the singularity is reached, machines that have become autonomous and superintelligent may no longer have to depend on us to gather energy, repair, build and improve themselves.

In the coming years, even before the singularity is reached, intelligent machines will continue to spread in all social structures, in economic and military systems, in transport, in energy production systems, in the food and manufacturing industry. Therefore, the idea of being able to unplug machine should they engage in malicious behavior should be considered very carefully, for our very survival. Imagine what would happen today if all the computers on the planet were shut down. The consequences would be catastrophic for humanity. Conversely, a superintelligence that were to come into conflict with humans to obtain the energy it needs would have total control of human systems and would take a fraction of a second to plan a winning strategy.

This line of thought, in which intelligent machines evolve as autonomous entities up to surpassing human capabilities, inevitably leads to catastrophic scenarios in which the human species is destined to become extinct due to the technology it has created. Scenarios like this have been featured in several science fiction films, including *Terminator 2: Judgment Day, Transcendence*, and *Matrix*. Does that mean we are really doomed or are there other possible scenarios?

## 9. Toward immortality

Fortunately, there is another possible scenario: the integration between humans and machines. This possibility has been considered and discussed in depth by Kurzweil (2005) in his book “The singularity is near.”

In fact, robotic and computer technologies also act on human nature and, since several years, different types of microchips have been developed to be installed inside the body to compensate for defects of specific organs. Think of the pacemaker to restore the correct heart rhythm, the artificial retina to compensate for visual defects, the subcutaneous micro-pumps to inject the right amount of insulin to diabetics, different types of computer-controlled robotic prostheses, and microchips implanted in the brain to prevent epileptic attacks or attenuate effects of Parkinson's disease.

These technologies will also evolve exponentially and, if today they are mostly used to compensate for defects, in the future they will also be adopted to enhance our sensory, motor and memory capacities. Robotics and bionic engineering will make it possible to enhance the body with more resistant and long-lasting artificial organs and limbs, while nanotechnology will develop biocompatible neural interfaces to enable neurons to communicate with computing devices.

At the same time, neurobiology will have evolved to the point where we understand in detail how the human brain works. Already today, high-resolution scanning techniques have made it possible to build a detailed map of the neuronal connections of a human brain (Caruso, 2016). These studies will be essential to understand the functioning of each neural circuit. Other projects^5^ are developing detailed models of neural circuits to accurately simulate the brain.

Once the functioning of the human brain is fully understood, nanotechnology, robotics and artificial intelligence will open up an unprecedented path, which will make it possible to build memory expansions, amplify sensory capabilities in order to perceive new signals, such as ultrasound or infrared, or offer the ability to add new sensory capabilities, in order to perceive, for example, radio signals or magnetic fields.

When a biological brain becomes able to communicate with a computer, it will be possible to implant coprocessors in the cerebral cortex for assisted recognition of objects, faces, voices, smells, tastes, etc. Furthermore, direct communication between the brain and the computer will make it possible to transmit thoughts, sensations and commands through wireless networks, creating a sort of “telepathic connection” with other individuals (human or artificial) and with computerized objects operating in the physical world.

We can imagine the possibility of sharing visual and sensory experiences with our distant friends: the images taken from our eyes would be processed by microchips implanted in our body to be transmitted to the corresponding devices installed in our friends' bodies, which would convert them to be “visualized” by their brain.

But this would only be the beginning of a new epochal revolution. The next step would be to gradually replace biological neurons with synthetic neurons to digitize the entire brain, thus overcoming the problem of biological degradation. American philosopher Bostrom (2016), director of the *Future of Humanity Institute* at the University of Oxford, argues that the transition between a biological and a synthetic brain would be almost imperceptible if the replacement of neurons took place gradually.

The benefits of owning a digital brain would be enormous. Considering that the response times of electronic components are in the order of nanoseconds, against the milliseconds of biological neurons, processing and learning times would be millions of times lower than those of a biological brain. But the most important aspect is that a digital brain would allow the information contained in it to be saved in one or more remote memories, so that they can be reloaded later in the event of hardware damage. This operation is referred to as “mind uploading” and is the key to achieving immortality.

Another advantage of a digital mind, typical of all digital products, is the possibility of transferring information at the speed of light. Just as a document can be scanned, transmitted to the other side of the world and reconstructed with a printer, in the same way, a digital intelligent being could be scanned, transmitted to another very remote place and reconstructed by a special advanced 3D printer, a kind of “materializer.”

Thanks to a digital mind and a replaceable synthetic body, humankind would make an unprecedented evolutionary leap, transforming itself into an immortal species, endowed with superintelligence and collective learning ability. The ability to transmit information at the speed of light would allow the new species to colonize the solar system, explore new worlds and expand into the universe.

## 10. The crossroads

From the analysis presented above, based on the exponential progress of artificial intelligence, only two possible extreme scenarios seem to emerge:

1. **Self destruction**. The first scenario is catastrophic, indicating that an uncontrolled evolution of machines could lead to the development of a superintelligence which, once it becomes autonomous, would seek to self-sustain and expand at an exponential rate, requiring ever greater energy resources. This would lead to a situation of conflict with the human species, which would have little chance of surviving against a superintelligence that has gained control of all human systems over the years.
2. **Immortality**. The second scenario assumes that the evolution of technology also has an impact on humankind, that will not stand still and watch intelligent machines overtake, but will exploit the possibilities offered by nanotechnology, robotics and artificial intelligence to gradually transform itself into a new species with synthetic bodies and digital brains. No longer subject to biological degradation and having the possibility of saving and restoring his mind, the human being will make an evolutionary leap, becoming himself an immortal superintelligence destined to expand into the universe.

Although the second scenario offers humanity a hope of survival from cosmic catastrophes, such as a new ice age, the impact of large meteorites, or the death of the Sun, which would lead to a certain extinction of the human species, the idea of transforming ourselves into synthetic beings is not welcomed by many people, who would prefer to stop the progress of technology.

Unfortunately, however, evolution is a process bigger than us, which cannot be stopped. Kelly (2010) in his book “What technology wants” expresses this concept very clearly, explaining how technology is like a living organism in constant evolution, with its own needs and unconscious tendencies. We can only observe it to better predict its movements and prepare for what is to come.

Someone could suggest halting technological evolution through restrictive policies that, for example, prohibit further research on artificial intelligence or oblige machine builders to set limits on the maximum levels of intelligence of future artificial brains. In reality, these solutions would be even more dangerous, because they would give rise to occult and unauthorized research activities whose results would be less predictable and even more risky for humans.

Others might think of halting the process through terrorist actions, but they would only slow it down temporarily, unless destroying the entire human race, which would only speed up the advent of the first scenario.

## 11. Conclusions

The exponential progress followed by computers and artificial intelligence in the last 80 years leads to predict that machines will reach a generalized human intelligence around 2030 and continue to evolve at an exponential rate, far surpassing human capabilities in the following years.

The considerations presented in this article seem to suggest that in the near future humanity is destined to find itself at a crossroads that bring only to two extreme and opposite scenarios: on the one hand, extinction, on the other, immortality.

A general law that has been observed for all living species that have appeared on this planet is that those who do not adapt to environmental changes die out. Similarly, if the human species will not be able to adapt to the technological changes that it has itself generated, it will be destined for extinction, to make room for another species of superintelligent artificial beings.

If, on the other hand, humankind will be able to adapt and change thanks to those same disruptive technologies, to merge with the ongoing evolutionary process and become a superintelligence itself, then it will have the key to become immortal and colonize the universe.

Predicting which of the two paths will be taken is not an easy undertaking, since it depends on how we will be able to exploit the potential that new technologies offer us. However, one thing is certain: this century will be full of surprises.

## Author contributions

The author confirms being the sole contributor of this work and has approved it for publication.

## Footnotes

^1^“New Navy Device Learns by Doing,” New York Times, July 7, 1958.

^2^ http://www.image-net.org/

^3^AIVA. https://www.aiva.ai/.

^4^Synthesia, 2017. https://www.synthesia.io/.

^5^Human Brain Project: https://www.humanbrainproject.eu/en/.
